# *TP53* p.R337H is a conditional cancer-predisposing mutation: further evidence from a homozygous patient

**DOI:** 10.1186/1471-2407-13-187

**Published:** 2013-04-09

**Authors:** Juliana Giacomazzi, Simone Selistre, Juliana Duarte, Jorge Pinto Ribeiro, Paulo JC Vieira, Gabriel de Souza Macedo, Cristina Rossi, Mauro Czepielewski, Cristina Brinkmann Oliveira Netto, Pierre Hainaut, Patricia Ashton-Prolla

**Affiliations:** 1 Genomic Medicine Laboratory, Experimental Research Center, Hospital de Clínicas de Porto Alegre (HCPA), Porto Alegre, Brazil; 2 Post-Graduate Program in Medicine: Medical Sciences, Universidade Federal do Rio Grande do Sul (UFRGS), Porto Alegre, Brazil; 3 Pediatric Oncology Service, HCPA, Porto Alegre, Brazil; 4 Radiology Service, HCPA, Porto Alegre, Brazil; 5 Exercise Pathophysiology Research Laboratory and Cardiology Division, HCPA, Porto Alegre, Brazil; 6 Post-Graduate Program in Genetics and Molecular Biology, UFRGS, Porto Alegre, Brazil; 7 School of Medicine, UFRGS, Porto Alegre, Brazil; 8 Department of Internal Medicine, Faculty of Medicine, UFRGS, Porto Alegre, Brazil; 9 Service of Endocrinology, HCPA, Porto Alegre, Brazil; 10 Service of Medical Genetics, HCPA, Porto Alegre, Brazil; 11 International Prevention Research Institute, Lyon, France; 12 Departamento de Genética, UFRGS e Serviço de Genética Médica e Centro de Pesquisa Experimental, Hospital de Clínicas de Porto Alegre, Rua Ramiro Barcelos, 2350, 90035-903 Porto Alegre, RS, Brazil

## Abstract

**Background:**

Adrenocortical carcinomas (ACCs) are among the most common childhood cancers occurring in infants affected with the Li-Fraumeni and Li- Fraumeni-like (LFS/LFL) syndromes, which are caused by dominant germline mutations in the *TP53* gene. In Brazil, a particular mutation, occurring in the tetramerisation domain of the gene, p.R337H, is exceedingly common due to a founder effect and is strongly associated with ACC. In this report, we describe the phenotype and long-term clinical follow-up of a female child diagnosed with ACC and homozygous for the *TP53* p.R337H founder mutation.

**Case presentation:**

At age 11 months, the patient was diagnosed with a virilising anaplastic adrenal cortical tumour, which was completely excised without disturbing the adrenal capsule. Family history was consistent with an LFL tumour pattern, and genotyping identified the *TP53* p.R337H mutation in both alleles in genomic DNA from lymphocytes and fibroblasts. Haplotype analysis confirmed the occurrence of the mutation in the same founder haplotype previously described in other Brazilian patients. No other germline or somatic *TP53* mutations or rearrangements were identified. At age 9 years, the child was asymptomatic and had no evidence of endocrine derangements. Full body and brain magnetic resonance imaging (MRI) failed to detect any suspicious proliferative lesions, and cardiopulmonary exercise testing results were within the normal reference for the child’s age, ruling out a major exercise capacity deficiency.

**Conclusion:**

This is the first clinical and aerobic functional capacity documentation of a patient who carries two mutant *TP53* alleles and no wild-type allele. Our results support the hypothesis that *TP53* p.R337H, the most common *TP53* mutation ever described in any population, is a conditional mutant. Furthermore, our observations over a long period of clinical follow-up suggest that *TP53* p.R337H homozygotes do not have a more severe disease phenotype than do heterozygote carriers of the same mutation. Patients with the homozygous *TP53* p.R337H genotype will require careful surveillance for lifetime cancer risk and for effects on metabolic capacity later in life.

## Background

Li-Fraumeni syndrome (LFS; OMIM# 151623) is an autosomal dominant disorder that predisposes carriers to multiple, early-onset cancers that are difficult to treat and often lethal. The most common childhood and adolescent cancers occurring in the classical form of the syndrome are soft-tissue sarcomas and osteosarcomas. Leukaemia and brain tumours occur throughout childhood and young adulthood, whereas adrenal cortical carcinomas (ACC) and choroid plexus carcinoma occur primarily in infancy. In young adults, breast cancer is the most common malignancy. Other tumours observed in LFS patients include colorectal, lung, gastric, pancreatic and prostate cancers, as well as melanoma and lymphoma [1-3]. Variant forms of the disease, observed in families with tumours of the LFS spectrum, which resemble but do not meet the strict criteria for LFS syndrome, have been collectively named Li-Fraumeni-like (LFL) syndrome [1,4].

The only known genetic defect associated with LFS and LFL is the inheritance of a mutation in the tumour suppressor gene *TP53*[4,5]. Mutations occur in up to 80% of the classical forms of LFS. *TP53* encodes a 53 kD nuclear phosphoprotein, p53, that acts as a growth suppressor transcription factor, which is inactivated by somatic mutations in many forms of cancer. The most frequent *TP53* germline mutations are missense substitutions that cluster in highly conserved regions of the DNA-binding domain of the protein (codons 125-300), with hotspots at highly mutable CpG motifs that are also found in somatic mutations in sporadic cancers. In Brazil, a particular mutation has been reported in a significant proportion of the LFS families presenting germline *TP53* mutations. This mutation, p.R337H (c.1010 G > A, genomic nucleotide number 16901; CGC to CAC at codon 337), occurs in exon 10 and was initially identified in Brazilian children with ACC and no documented familial history of other cancers [6,7]. The arginine residue at codon 337 is part of an alpha-helix motif involved in p53 oligomerisation. Structural studies on the p53 oligomerisation domain have shown that replacement of arginine by histidine disrupts oligomerisation in a pH-dependent manner, making the domain unable to oligomerise in conditions of slightly elevated pH [8,9]. Although biological dependence upon pH has not been demonstrated thus far, it is plausible that the p.R337H mutant protein operates as a conditional mutant that loses its function only in cells undergoing a small increase in intracellular pH. This type of change may be occurring in cells undergoing programmed cell death during developmental tissue remodelling, such as in the peri-natal adrenal cortex.

Subsequent to the initial studies on childhood ACC, studies of families with LFS traits have shown that the mutation predisposes individuals to a wide range of cancers. Similar to the canonical forms of LFS, the most common cancers in p.R337H carriers are pre-menopausal breast cancer and sarcoma before age 45 [10-12]. However, the penetrance of the disease in p.R337H carriers appears to be significantly lower than in carriers of germline *TP53* mutations occurring in the DNA-binding domain. *TP53* haplotyping of 12 apparently unrelated p.R337H-positive families showed that the mutation occurred on the same allele, demonstrating a founder effect [12,13].

Population-based studies indicate that the carrier rate in southern and southeastern Brazil is of approximately 0.3% [14,15]. Assuming Hardy-Weinberg equilibrium, mutant homozygotes (i.e., individuals who inherited one mutant allele from each parent) are predicted to occur with a frequency of approximately 1 in every 455.000 live births based on this relatively high carrier prevalence.

Studies in *TP53*-deficient mice have shown a reduced exercise capacity associated with a lower mitochondrial density in skeletal muscle [16-18]. *TP53*-deficient mice did not respond to a 5-week aerobic training protocol, indicating that p53 is required to complete the adaptive changes in aerobic metabolism that are necessary for increasing exercise capacity in response to training [17]. However, no information is currently available on the impact of *TP53* deficiency on the exercise capacity of patients with germline *TP53* mutations. Here we report the diagnosis, follow-up and monitoring of exercise capacity in a young patient homozygous for the germline *TP53* p.R337H mutation.

## Case presentation

A previously healthy female child of European (Portuguese and Spanish) descent was referred to a paediatrician at 11 months of age with a history of increased appetite, significant weight gain in the past three months, and signs of virilisation. Review of the family history revealed second- and third-degree relatives diagnosed with cancer consistent with a LFL tumour pattern. Both parents were healthy, with no personal history of cancer, and were unrelated. On admission, the patient weighed 14.550 kg (> the 95th percentile) and measured 83 cm (> the 95th percentile). Blood pressure was 130/90 mmHg, and physical examination revealed a round “moon-like” face, excess facial and body hair, pubarche and clitoromegaly, facial acne and an abdominal mass on palpation.

Laboratory evaluations showed normal serum sodium, potassium, calcium, phosphorus and creatinine. Hormonal evaluations showed very high levels of androgens: dehydroepiandrosterone sulphate (DHEAS) > 1000 μg/dL (nl: 2-274 μg/dL), dehydroepiandrosterone > 30 ng/ml (nl: < 2,5 ng/ml), testosterone 9.61 ng/dL (nl: < 0,05 ng/dL), androstenedione > 10 ng/dL (nl: < 0,5 ng/ml), 17-alpha-hydroxyprogesterone 8,88 ng/ml (nl: < 1,0 ng/ml), progesterone 4803 pg/ml (nl: < 800 pg/ml), morning cortisol 37 μg/dl (nl: 4,3-22,4 μg/dl), and midnight cortisol 32 μg/dl (nl: < 1,0 μg/dl). Urinary free cortisol was normal: 80 μg/24 h (nl: 20 – 90 μg/24 h), and ACTH was undetectable. Bone age was assessed using the Greulich-Pyle method and was found to be 24 months (SD = 2.4 months) despite a chronological age of 14 months. Abdominal computerised tomography identified a heterogeneous adrenal mass (4.5 × 3.4 × 3.0 cm), which was excised without disturbing the adrenal capsule. Surgical margins were negative, and histopathological examination of the tumour tissue confirmed the diagnosis of an anaplastic adrenal cortical tumour (4.5 × 4.0 × 2.8 cm) weighing 27 gram.

**Figure 1:**
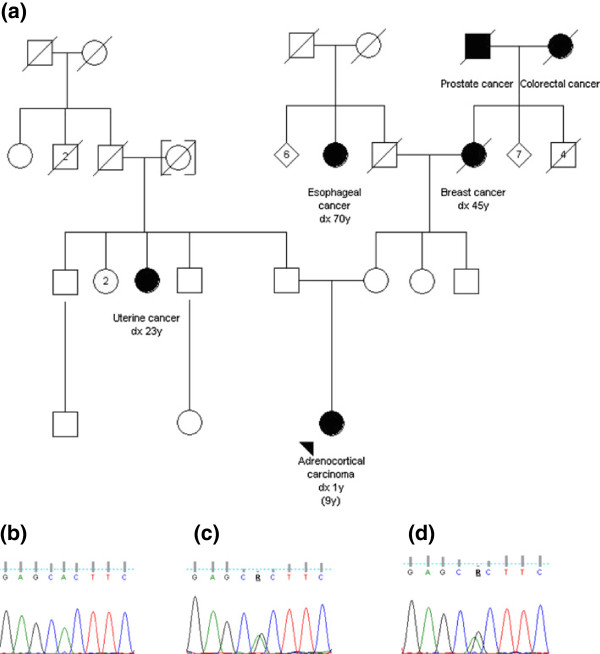
**Pedigree of the homozygous** ***TP53* p.R337H/p.R337H patient and** ***TP53* Exon 10 sequencing results from the proband and parents.** Dx: age at diagnosis; WT: wild-type (**a**) Pedigree of the homozygous *TP53* p.R337H/p.R337H patient. Relatives affected by cancer are shown with blackened symbols; the arrow indicates the proband; current age is indicated in parenthesis. *TP53* exon 10 sequencing results from the proband (**b**) demonstrating homozygosity for the A allele at genomic nucleotide number 16901 and from her parents (**c**, **d**) showing heterozygosity at the same nucleotide position.

At the age of 6 years, the patient was recruited for a *TP53* mutation prevalence study, which was offered to all patients diagnosed or treated for paediatric tumours of the LFS cancer spectrum at Hospital de Clínicas de Porto Alegre from 1998 to 2010 (IRB# 08022). The patient was found to be homozygous for a germline *TP53* mutation (p.R337H) (detailed genotypic analysis is described below). The patient is under follow-up with paediatric oncology, endocrinology, and cancer genetics specialists. Ninety-six months after the diagnosis of the ACC (at age 9 years), the patient appears healthy and has adequate cognitive and psychomotor development. There is currently no clinical or laboratory evidence of endocrine derangements. After diagnosis, the patient was followed by the paediatric oncology team through age 8 years, according to the NCCN [19] guidelines. Full body and brain magnetic resonance imaging (Figure 1) and cardiopulmonary exercise testing (Table 1) were performed at age 8 years and 8 months. MRI failed to detect any suspicious proliferative lesions, and the results of the cardiopulmonary exercise test were within the normal reference for 9-year-old girls; these results indicated that there was no major exercise capacity deficiency in this patient. A detailed description of the evaluations performed is presented below.

**Table 1:** **Anthropometric data and results of the cardiopulmonary exercise test for the *TP53 *p.R337H homozygous patient at the age of 8 years and 8 months**

**Variable**	**Measured**	Percentage of predicted*
Weight (kg and %)	37.5	114
Height (cm and %)	144.5	105
Body mass index (kg/cm^2^ and %)	17.9	96
Peak power output (W and %)	80	101
Peak heart rate (bpm and %)	164	88
Peak VO_2_ (mL/kg.min and %)	35.9	85
V_E_/VCO_2_ slope (ratio and %)	25.2	78
Peak respiratory exchange ratio (ratio and %)	1.11	97
Peak O_2_ saturation (%)	98	NA
Anaerobic threshold (mL/kg.min and %)	24.9	96
Peak stroke volume (mL/min)	46	NA
Peak cardiac output (L/min)	7.62	NA

Legend: *Predicted values for girls on the cycle ergometer, according to Ten Harkel et al. 2011; NA: reference value not available; bpm: beats per minute; VO_2_: oxygen uptake; V_E_: minute ventilation; VCO_2_: carbon dioxide production.

### Evaluation of functional capacity

At the age of 8 years and 8 months, the patient was submitted to maximal exercise testing on an electromagnetically braked cycle ergometer (ER-900, Ergoline, Jaeger, Würzburg, Germany) at 60 revolutions per min. She was not receiving any medication. Hormonal evaluations showed undetectable androgens, and bone age corresponded to chronological age. The patient first exercised for 3 min with no load; the work rate was then increased by 10 W per min until volitional fatigue, indicated by the inability to maintain the pedalling rate above 40 revolutions per min. A twelve-lead electrocardiogram (Nihon Khoden Corp.,Tokyo, Japan) was continuously monitored, and expired gases were collected breath-by-breath by a computerised gas analyser (Oxycon Delta, VIASYS, Healthcare GmbH, Würzburg, Germany). Peak values for oxygen uptake and respiratory exchange ratio are reported as the highest 20 s mean values. The anaerobic threshold (also referred to as the first ventilatory threshold) was determined by review of the gas exchange curves; the anaerobic threshold is defined as the oxygen uptake at which the ventilatory equivalent for oxygen increased systematically without an incremental change in the ventilatory equivalent for carbon dioxide [20]. The per minute ventilation/carbon dioxide output slope was calculated using a linear regression analysis based on all points of the incremental exercise [20]. Before and during the exercise tests, cardiac output and stroke volume were estimated non-invasively by impedance cardiography (PhysioFlow PF07 Enduro, Manatec Biomedical, Paris, France) as previously described [21]. Arterial oxygen saturation was measured by finger oximetry (Takaoka Oxicap, São Paulo, Brazil).

The results of the cardiopulmonary exercise test, including the percentage of the predicted values for girls [22], are presented in Table 1. The test was stopped due to fatigue, with the peak heart rate and the peak respiratory exchange ratio compatible with maximal effort. Compared to the reference values, the patient presented peak power output, peak oxygen uptake, and anaerobic threshold consistent with preserved exercise capacity (85 to 101% of predicted). The minute ventilation/carbon dioxide output slope indicated normal ventilatory efficiency. Stroke volume and cardiac output increased appropriately during the incremental exercise test [23].

### Genetic analyses

**Figure 2:**
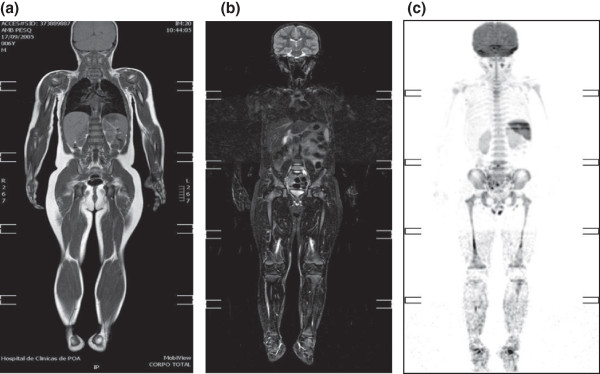
**Whole body magnetic resonance imaging of the *TP53 *p.R337H homozygous patient.** (**a**) Coronal plane T1 Turbo Spin Echo (TSE) weighted image, (**b**) coronal plane Turbo Short TI Inversion Recovery (STIR) image and (**c**) coronal plane image recorded after diffusion-weighted imaging (DWI) on the axial plane with further maximum intensity projection (MIP) reconstruction. Imaging: Brain and whole body magnetic resonance imaging studies were conducted using a 1.5 Philips Achieva (Philips Healthcare, Latham, NY, USA) series scanner following the protocols described [25,26], with modifications. The diffusion-weighted imaging with body background suppression protocol using a free-breathing technique, as described [27], was applied. In brief, T1 Turbo Spin Echo (T1 TSE) weighted images (T1) and Short TI Inversion. Recovery (STIR) were performed on the coronal plane, and diffusion-weighted imaging (DWI) was performed on the axial plane with further maximum intensity projection (MIP) reconstruction on the coronal plane. We used the body coil and asked for the breath to be held for the thorax and abdomen image acquisitions. Restricted diffusion was observed in the area corresponding to the bone marrow of the lower limbs but followed the expected pattern for age, as described previously [28].

Genomic DNA was obtained from (a) peripheral lymphocytes and fibroblasts using the Ilustra™ blood genomic Prep Mini spin Kit (GE Healthcare, Madison, WI, USA) as described by the manufacturer and from (b) formalin-fixed paraffin-embedded tumour tissues using the QIAmp DNA FFPE Tissue Kit (Qiagen, Hilden, Germany). DNA from lymphocytes was screened for the *TP53* p.R337H mutation with allele-specific TaqMan® probes (Applied Biosystems, Foster City, CA, USA) using a MX 3000P™qPCR System - Stratagene (Agilent Technologies Inc., Santa Clara, CA, USA) and analysed using the Stratagene MxPro qPCR Software. Presence of the mutation was confirmed by PCR-RFLP using the restriction enzyme *HhaI*[14] and by bidirectional gene sequencing of exon 10 using an ABI PRISM 3130XL Genetic Analyzer (Applied Biosystems, Foster City, CA, USA) following the sequencing protocol described in the *IARC TP53* database [24]. All genotyping results were confirmed in at least two independent analyses. The only nucleotide detected at position 17588 was A (CAC at codon 337, homozygote for the mutant allele) in both the genomic DNA from peripheral lymphocytes and fibroblasts and in the DNA extracted from microdissected, formalin-fixed, paraffin-embedded ACC tissues. Both the father and mother were found to be heterozygous *TP53* p.R337H carriers (Figure 2). Direct sequencing of the entire *TP53* coding region (exons 2-11) of the index case and the parents did not detect other germline mutations. Allele-specific PCR (ASO PCR) for *TP53* SNP179 identified the proband as well as both parents as carriers of the p.R337H Brazilian founder allele described by Garritano et al. (2010) [12]. The presence of the Brazilian founder p.R337H haplotype (A3) was verified in mutation-positive samples, as previously described [12], by both ASO-PCR and Nested-PCR used to analyse SNP28 (rs9894946) from a panel of 29 intragenic SNPs. Because this SNP is identical in haplotypes A1 and A3, all cases were further genotyped for SNP15 (rs1642785) and SNP18 (rs1800370) by direct sequencing. Multiplex ligand probe amplification (MLPA), used to assess the presence of *TP53* gene deletions, was performed in the index case using genomic DNA from leukocytes and fibroblasts and the SALSA P056 kit (MRC, Amsterdam, The Netherlands), according to the manufacturer’s instructions. No *TP53* deletion was identified in the germline. With these results, a homozygous genotype at position 16901 was inferred, although uniparental disomy could not be definitively excluded. We were unable to confirm whether one of the mutant alleles was lost in the tumor, due to limited availability of tumoral tissue.

## Discussion

We describe a patient who is a homozygous carrier of a germline *TP53* mutation, p.R337H, having inherited the same mutant allele from both parents. The patient, currently aged 9 years, developed ACC at age 11 months and has been under clinical follow-up for other health outcomes, including cancers of the Li-Fraumeni spectrum, since that time. At age 9 years, the patient was healthy, with normal development, normal cardiopulmonary/exercising capacity and no suspicious proliferative lesions detectable using standard whole-body MRI.

In western Europe, it has been estimated that germline *TP53* mutations spontaneously occur at a rate of 1:5,000 births [29]. Approximately 50% of mutation carriers develop cancer by age 30. Carriage of two mutant alleles in the classic DNA-binding domain region of the gene (either through *de novo* mutation on both alleles or through inheritance of a mutant from both paternal and maternal sides) is therefore predicted to be an extremely rare event; indeed, there have been no reports of such an event. In Brazil, however, the existence of a founder mutation, p.R337H, which is present in approximately 0.3% of the dense population in the southern and southeastern regions of the country, make the occurrence of homozygosity for germline *TP53* mutations more likely than anywhere else in the world. Prior to this report, homozygosity for p.R337H had been observed in one subject in a study of 55 Brazilian paediatric and adult patients with apparently sporadic ACC. That patient, a girl diagnosed with ACC at 12 months of age and whose parents were unaffected carriers, was healthy and had not developed another cancer at age 10 years [30], Latronico and Fragoso, personal communication 2011].

Several studies have shown that germline mutations in cancer predisposition genes may have a dose effect, resulting in a more severe phenotype in homozygotes than in heterozygotes. For example, the heterozygous state of the *CHEK2* 1100delC variant, which predisposes carriers to breast cancer, is associated with an OR of 1.5-3.0 (corresponding to a lifetime risk of 20-25%), while the homozygous state is associated with a greater than fourfold increase in the lifetime risk compared with the general population [31,32]. Furthermore, several cancer-predisposing mutations in tumour-suppressor genes display different phenotypes in heterozygotes and homozygotes. This is the case for mutations in the *PALB2*, *BRIP1* and *ATM* genes, where heterozygotes have an increased lifetime risk of breast cancer, and homozygotes are diagnosed with multisystemic genetic syndromes (Fanconi’s anaemia for the first 2 genes and ataxia-telangiectasia for the latter). There are also situations where homozygous and heterozygous states for a mutation in the same gene are each associated with different genetic syndromes, such as the Lynch (LS) and Childhood Cancer (CCS) syndromes associated with heterozygous and homozygous germline mutations in the MMR genes [33,34].

In the present case, our observations do not support the hypothesis that inheritance of two mutant *TP53* alleles may lead to a compound phenotype with increased risk for early onset cancer. It is possible that the absence of cumulative effects is due to the particular structural properties of the p.R337H mutant protein. Based on the structural analysis of a peptide encoding the oligomerisation domain, it has been shown that replacement of arginine by histidine at position 377 perturbs the formation of a hydrogen bond that links R377 on one p53 monomer to D352 on another p53 monomer, thus forming a dimer [8]. At pH 7, histidine at position 377 retains the capacity to donate a H-bond. However, at pH 8, this capacity is lost, thus preventing dimerisation at slightly elevated pH levels. In the present case, we speculate that the proteins encoded by the two mutant alleles can form dimers in neutral pH conditions, which also occurs between one mutant and one wild-type monomer in patients who inherit only one mutant p.R337H allele. However, upon a small pH increase, the hydrogen bonds between the monomers, which form the homodimer consisting of the two mutant proteins, would be expected to break.

Recent experimental studies have shown that p53 plays a critical role in controlling cell bioenergetics and, specifically, mitochondrial metabolism [18]. In the mouse, lack of p53 leads to impaired cardiorespiratory fitness and loss of aerobic competence. Mice lacking functional p53 show a decrease in maximum exercise capacity and are less responsive to the effects of training than their p53-competent counterparts [16,17]. However, such effects have not been documented in humans thus far. The results of the cardiopulmonary exercise test performed here indicate that, despite carrying two *TP53* mutant alleles, our patient has preserved functional capacity, as demonstrated by peak power output, peak oxygen uptake, and an anaerobic threshold within the limits of normality [21]. Moreover, ventilatory efficiency and hemodynamic responses to exercise were also normal. If mitochondrial abnormalities were to be present in our patient, we would expect to observe an early anaerobic threshold, reduced peak oxygen uptake, and ventilatory inefficiency. Because individuals with metabolic abnormalities, such as patients with McArdle’s disease, may present hyperventilation during exercise without blood lactate accumulation [35], a preserved ventilatory anaerobic threshold, as observed in our patient, may not assure normal muscle oxidative metabolism. However, normal maximal exercise capacity and a normal anaerobic threshold are strong indicators of preserved muscular oxidative capacity. In healthy individuals, there is a strong association between muscle respiratory capacity and the anaerobic threshold [36]. Although an influence of previous or current androgen excess on energy metabolism in this patient cannot be excluded, such an influence is unlikely because cardiovascular function was assessed many years after the normalisation of the hormone levels and bone age results were normal. Therefore, the cardiopulmonary exercise test results for our patient indicate that inheritance of two mutant *TP53* p.R337H alleles does not appear to affect energy metabolism in humans by the age of 10 years.

## Conclusion

The current report is the first clinical description and documentation of aerobic functional capacity of a patient who carries two mutant *TP53* alleles and no wild-type allele. Our results support the hypothesis that *TP53* p.R337H, the most common *TP53* mutation ever described in any population, is a conditional mutant. Furthermore, our observations over a long period of clinical follow-up do not support the hypothesis that p.R337H homozygotes may have a more severe disease phenotype than heterozygote carriers of the same mutation. However, the particular genetic status of these patients will require careful surveillance for lifetime cancer risk and for effects on metabolic capacity later in life.

### Consent

Written informed consent was obtained from the parents of the child for publication of this case report and accompanying images. A copy of the written consent is available for review by the Series Editor of this journal.

## Competing interests

The authors declare that they have no competing interests.

## Authors’ contributions

JG carried out the molecular genetic studies, was involved in patient recruitment and in all steps of data analysis and manuscript writing. SS, MC, CR and CBON provided clinical data, were directly involved in the clinical follow-up and helped to draft the manuscript. GSM provided laboratory support, carried out fibroblast cultures and genetic studies and helped to draft the manuscript. JD carried out and interpreted the imaging studies, and helped to draft the manuscript. JPR and PJCV carried out and interpreted the cardiovascular performance studies, and helped to draft and revise the manuscript. PH was directly involved in the conception and design of the case report and critically reviewed the manuscript. PA-P was directly involved in the conception and design of the case report, participated in the clinical follow-up of the patient and interpretation of clinical and laboratory data, and coordinated manuscript writing. All authors read and approved the final manuscript.

## Pre-publication history

The pre-publication history for this paper can be accessed here:

http://www.biomedcentral.com/1471-2407/13/187/prepub
